# Modeling of Electrical Conductivity for Polymer–Carbon Nanofiber Systems

**DOI:** 10.3390/ma15197041

**Published:** 2022-10-10

**Authors:** Sajad Khalil Arjmandi, Jafar Khademzadeh Yeganeh, Yasser Zare, Kyong Yop Rhee

**Affiliations:** 1 Department of Polymer Engineering, Faculty of Engineering, Qom University of Technology, Qom 371951519, Iran; 2 Biomaterials and Tissue Engineering Research Group, Breast Cancer Research Center, Department of Interdisciplinary Technologies, Motamed Cancer Institute, ACECR, Tehran 1125342432, Iran; 3 Department of Mechanical Engineering (BK21 Four), College of Engineering, Kyung Hee University, Yongin 17104, Korea

**Keywords:** carbon nanofiber, polymer nanocomposites, electrical conductivity, tunneling, interphase, parametric analysis, volume fraction, percolation

## Abstract

There is not a simple model for predicting the electrical conductivity of carbon nanofiber (CNF)–polymer composites. In this manuscript, a model is proposed to predict the conductivity of CNF-filled composites. The developed model assumes the roles of CNF volume fraction, CNF dimensions, percolation onset, interphase thickness, CNF waviness, tunneling length among nanoparticles, and the fraction of the networked CNF. The outputs of the developed model correctly agree with the experimentally measured conductivity of several samples. Additionally, parametric analyses confirm the acceptable impacts of main factors on the conductivity of composites. A higher conductivity is achieved by smaller waviness and lower radius of CNFs, lower percolation onset, less tunnel distance, and higher levels of interphase depth and fraction of percolated CNFs in the nanocomposite. The maximum conductivity is obtained at 2.37 S/m by the highest volume fraction and length of CNFs.

## 1. Introduction

The development of electronic technology has rapidly increased the need for materials with perfect mechanical and physical properties [1,2,3,4,5,6,7,8]. These polymer-based nanocomposites have the capability of meeting these requirements [3,6,9,10,11,12,13,14,15,16,17,18]. The use of different conductive fillers has been widely demonstrated to raise the conductivity and electrical properties. Nanocomposites are defined as a new categorized material, which consists of at least one nanoscale filler dimension [19]. Carbon nanofibers (CNFs) are favorable candidates compared to other nanoparticles with carbon structures since they possess a high aspect ratio (diameter per unit thickness) and low electrical resistivity [20,21,22,23]. Their large aspect ratio leads to producing a conductive network in polymer matrices, which changes the conductivity. The notable change in conductivity is seen at the percolation onset point due to the formation of a conductive network after percolation onset [9,24,25,26]. Many equations and models have been proposed in the literature to understand the network formation and conductivity of nanocomposites [27,28,29]. Although CNFs and carbon nanotubes (CNTs) have a close resemblance in terms of morphology and microstructure, their differences have not been widely reflected. In comparison to CNFs, CNTs are more influenced by van der Waals forces, which cause them to stay dispersed for a shorter period of time [30,31,32].

Electrons can be transferred between nanoparticles and the contact regions among nanofibers play an important role in electron tunneling [33,34]. In fact, a short tunneling length as the distance between two adjacent nanosheets can positively increase the conductivity of composites as a tunneling mechanism. The tunneling effect has been studied in many papers, and can affect the electrical conductivity of CNF–polymer composites (PCNFs) [35,36,37,38]. In addition, the interphase zones between nanofillers and the polymer matrix can influence the properties of PCNFs. The impacts of interphase regions on the performances of nanocomposites have been reported in many studies [39,40,41,42,43,44,45,46,47,48]. It was reported that interphase regions can change the percolation level to smaller CNF volume fractions. In other words, the interphase depth can change the percolation onset, the conductivity, and the mechanical properties of a PCNF [29,49,50]. However, there are no models for reflecting the effects of the tunneling mechanism and the interphase zone on the conductivity of a PCNF, although these factors can contribute to higher conductivity and a smaller percolation threshold in nanocomposites.

The main advantages and novelty of this study include the applications of interphase and tunneling terms to predict the conductivity of PCNFs. The developed model considers the impacts of important factors such as tunneling distance, filler volume fraction, interphase thickness, and critical volume fraction of nanofibers (percolation onset). The roles of the tunneling region and interphase in the percolation threshold are properly indicated by the developed equations. To evaluate the proposed equations, many experimented data are applied. Additionally, the influences of various factors on the conductivity are plotted to validate the suggested equations.

## 2. Model Development

**Figure 1:**
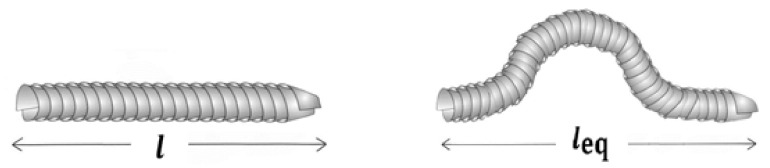
Straight and curved CNFs.

Percolation onset can be defined by using the aspect ratio of CNFs [51], as follows:

(1) $$\varphi_{p}\approx \frac{R}{l}$$

where “R” and “l” are the radius and length of CNF, respectively. A layer surrounding the CNF, which is defined as interphase, should be considered in Equation (1), because the interphase layer declines the percolation and quickens the networking [52]. So, the final equation for the percolation threshold is as follows:

(2) $$\varphi_{p}=\frac{15(R-2t)}{l}$$

where “t” is interphase depth. CNFs have lengths of a few microns with diameters of around 100 nm, which results in a high aspect ratio (length/diameter > 100). The effectiveness of carbon nanofibers is decreased by the waviness of a PCNF [51]. The function of waviness can be given by assuming an equivalent length ($l_{eq}$) for wavy nanofibers (Figure 1), as follows:

(3) $$u=\frac{l}{l_{eq}}$$

where a higher values of “u” exhibits further waviness, whilst u = 1 shows no curvature (straight nanofibers). Additionally, the electrical conduction of CNF can be decreased by curvature, which should be taken into consideration [51].

The impact of waviness on “$\sigma_{N}$”, as the natural conduction of a CNF, is expressed by:

(4) $$\sigma_{Nw}=\frac{\sigma_{N}}{u}$$

Additionally, a CNF and its surrounding interphase express the effective nanoparticles, which are able to determine the properties and performance of CNF-based composites.

The effective volume share of a CNF can be given [33] by:

(5) $$\varphi_{eff}=\frac{{\left(R+t\right)}^{2}(l/u+2t)}{R^{2}l/u}\varphi_{f}$$

where “$\varphi_{f}$” is total volume fraction of CNFs in a PCNF.

Moreover, some nanofibers are separated in a PCNF, while the remaining ones participate in networks above the percolation threshold. The percentage of percolated CNFs, which is computed by effective filler concentration and percolation onset, is described [33] as:

(6) $$f=\frac{\varphi_{eff}{}^{1/3}-\varphi_{p}{}^{1/3}}{1-\varphi_{p}{}^{1/3}}$$

**Figure 2:**
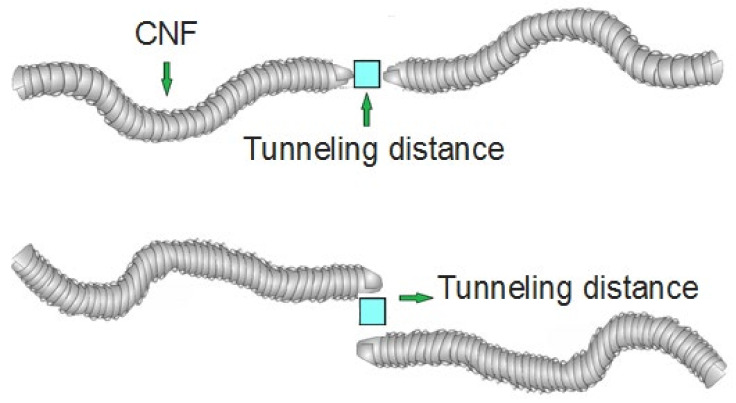
Placement of tunneling distance between adjacent nanofibers.

Deng and Zheng [53] suggested a model for conductivity of CNT-reinforced composites above percolation onset as:

(7) $$\sigma =\sigma_{0}+\frac{f\varphi_{f}\sigma_{N}}{3}$$

where “$\sigma_{0}$” is electrical conductivity of polymer matrix and “f”, “$\varphi_{f}$”, and “$\sigma_{N}$” were defined before. However, Equation (7) is not able to reveal the impacts of tunneling and interphase in the conductivity. Using the above equations, effective filler volume fraction, waviness, and the properties of interphase and tunnels can develop Equation (7) to forecast the conductivity of a PCNF as:

(8) $$\sigma =\frac{10^{-3}f^{3}\varphi_{eff}{}^{3}l\sigma_{Nw}}{d}$$

where “*d*” is the tunneling distance (Figure 2). Equation (8) expresses the electrical conductivity of a PCNF, assuming the roles of network fraction, effective volume fraction, filler conduction, and tunneling distance. Some terms, such as the oxygen functional groups, may affect the conductivity of a PCNF because it is widely known that the oxygen functional groups may reduce the electrical conductivity of CNF [54]. Additionally, the surface area resistance is effective on the conductivity [55].

The developed equations in this paper are accurate when a CNF is randomly embedded in the polymer medium. The type of polymer medium is not important, but the random orientation of CNFs in the polymer medium is important in our model.

## 3. Results and Discussion

### 3.1. Evaluation of Parameters

The effect of each factor on the conductivity of a PCNF is surveyed by the developed model, which is reported by 2D and 3D plots. Additionally, the average levels of factors such as R = 50 nm, t = 20 nm, l = 40 µm, u = 1.3, d = 5 nm, $\varphi_{f}$ = 0.02, and $\sigma_{N}=10^{4}$ S/m (on average) [56,57,58] are considered for all calculations.

**Figure 3:**
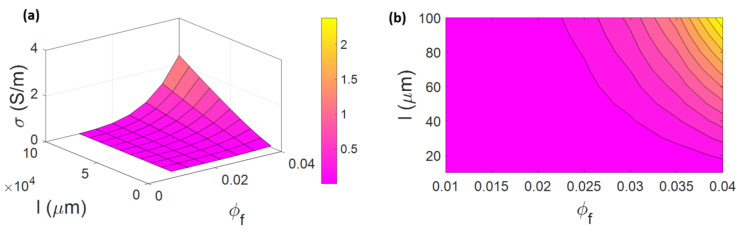
Impacts of “$\varphi_{f}$“ and “l“ on the conductivity by (**a**) 3D and (**b**) 2D diagrams.

The influences of “$\varphi_{f}$” and “l” on the conductivity are plotted in Figure 3. The higher ranges of “$\varphi_{f}$” and “l” cause the high levels of the electrical conductivity to be 2.37 S/m, whilst the lower levels of “$\varphi_{f}$” and “l” result in a lower conductivity. $\varphi_{f}$ < 0.0225 or *l* < 18 µm cause poor conductivity (near to 0). Additionally, the highest conductivity is obtained at $\varphi_{f}$ = 0.04 and l = 100 µm. CNFs are able to increase the electrical conductivity of nanocomposites due to their high inherent conductivity, which can reach to $\sigma_{N}=10^{4}$ S/m. There is a big difference between the conductions of CNFs and polymers. This considerable difference is due to the higher levels of CNF conduction than those of the polymer matrix. A high number of nanofibers can create the conductive networks in PCNF, which can accelerate the charge transfer and enhance the conductivity [59,60,61]. Furthermore, a high CNF length increases the aspect ratio, which can optimistically control the electrical conductivity of a PCNF. Larger nanofibers can participate in the network much more easily than shorter CNFs because longer nanoparticles decline the percolation threshold, as mentioned in previous equations. In addition, longer CNFs can simplify the transfer of electrons among nanofibers and form a conductive network promoting the conductivity [62,63,64]. Therefore, the advanced model properly handles the impacts of CNF concentration and length on the conductivity of a PCNF.

**Figure 4:**
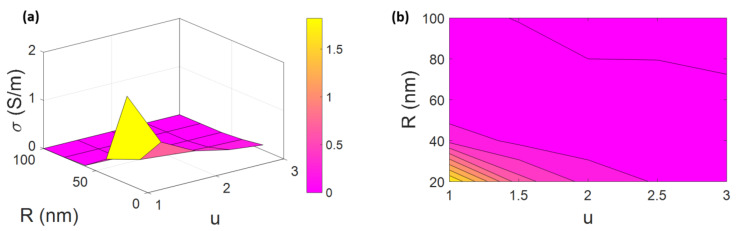
The stimuli of “u“ and “R “ on the conductivity by (**a**) 3D and (**b**) 2D designs.

Figure 4 shows the 3D and 2D diagrams of conductivity related to “u” and “R”. The lowest electrical conductivity of a PCNF is observed at the high levels of “u” and “R”. The maximum conductivity is achieved at 1.82 S/m at u = 1 and *R* = 20 nm, which means that the low values of CNF curvature and radius cause a high conductivity. Additionally, the composite is insulated at u > 2 and R > 80 nm.

CNF waviness, which is presented by “u”, negatively affects the effective volume fraction of nanofiber based on Equation (5). Additionally, “u” harmfully controls the CNF conduction, which can be seen in Equation (4). Higher levels of waviness negatively affect the effective length of nanofibers, and therefore the electrical conductivity of a PCNF. Additionally, a straight CNF causes a high range of conductivity [65,66]. Therefore, the suggested equations can properly show the effect of CNF curvature on the conductivity.

Thin nanofibers positively influence the percolation onset, effective volume fraction of CNFs, and the percentage of percolated CNFs based on Equations (2), (5), and (6), respectively. The lower values of CNF radius decrease the percolation onset, increase the effective volume fraction of CNFs, and increase the percentage of percolated CNFs, which results in the enhancement of conductivity. Furthermore, thicker nanofibers are not able to increase the aspect ratio and the effectiveness of CNFs, indicating that they cannot quicken the networking [36,67,68,69]. Hence, it is rational to achieve a higher conductivity by utilizing thinner CNFs, confirming the developed model.

**Figure 5:**
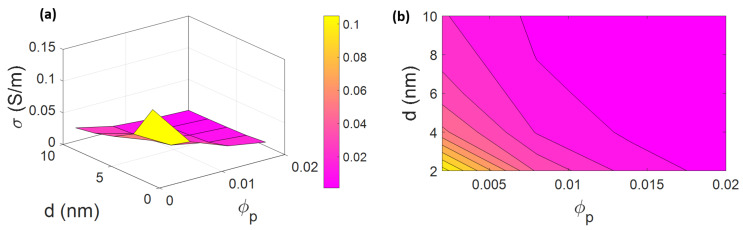
Theoretical conductivity by “$\varphi_{p}$“ and “d “: (**a**) 3D and (**b**) contour examples.

The illustrations of the electrical conductivity of PCNFs based on “$\varphi_{p}$” and “d” are presented in Figure 5. The best results are witnessed at the lowest levels of “$\varphi_{p}$” and “d”, which is calculated as about 0.1 S/m, while the lowest conduction is acquired at the highest values of the mentioned parameters. The poor electrical conductivity at 0 is seen at $\varphi_{p}$ > 0.0175 at all ranges of tunneling distance.

A low percolation threshold demonstrates that nanofibers can form dense networks throughout the nanocomposites (Equation (6)) [70,71]. So, the percolation onset can play an important role in the conductivity. The minimum number of nanofibers needed for creating a contiguous network in the nanocomposites is presented as “$\varphi_{p}$”. The percolation onset has an opposite impact on the conductivity of composites, which has been reported in previous studies [27,72,73]. In fact, a lower level of “$\varphi_{p}$” can expand the CNF network, whereas the charge transfer cannot be increased by a higher value of this term. This explanation justifies the predictions of conductivity at various ranges of percolation threshold.

A separation length between nanofibers defines the tunneling distance, which manages the behavior of CNFs for percolating and networking. The electrons are transferred between nanofibers by a tunneling effect. CNFs are capable of forming percolated networks and transferring charges when the distance between the nanofibers is lower than a critical value. In fact, a low level of tunneling distance enhances the electrical conductivity of PCNFs, which indicates the positive influence of the tunneling effect on the conductivity [35,36,74]. However, a longer tunneling distance leads to poorer transference of electrons or insulation, verifying the calculations.

**Figure 6:**
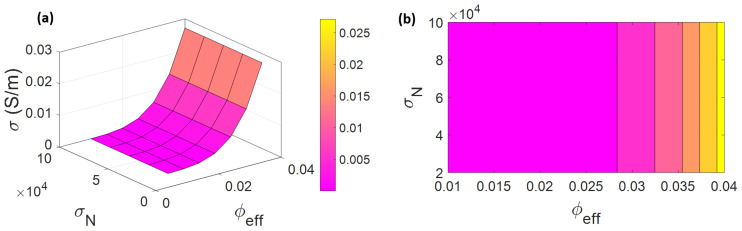
(**a**) Three-dimensional and (**b**) two-dimensional plots for the conductivity at various levels of “$\varphi_{eff}$” and “$\sigma_{N}$ ”.

The variations of conductivity by different levels of “$\varphi_{eff}$” and “$\sigma_{N}$” are indicated in Figure 6. The best conductivity is obtained by the highest ranges of “$\varphi_{eff}$” and “$\sigma_{N}$”, whilst the low levels of electrical conductivity are detected by the lowest values of these parameters. The highest conductivity for PCNF is acquired as 0.027 S/m by $\varphi_{eff}$ = 0.04, whilst $\varphi_{eff}$ < 0.028 leads to poor conductivity near to 0, which shows that CNF’s effective volume share has a significant effect on the conductivity.

“$\varphi_{eff}$” controls the electrical conductivity of PCNFs and higher levels of this parameter increase the conductivity because it shows the positive participation of interphases and CNFs in the networks. Additionally, long and thin CNFs and thick interphases cause a high level of CNF effective volume share based on Equation (5). In addition, a high level of “$\varphi_{eff}$” increases the “f”, which is seen in Equation (6). In other words, higher ranges of CNF effective volume share raise the fraction of networked nanoparticles, which directly affects the electrical conductivity [62,75,76]. Although high “$\varphi_{eff}$” raises the conductivity, the “$\sigma_{N}$” factor is not able to affect the conductivity. In fact, CNF effective volume share directly handles the conductivity, but CNF conduction cannot manipulate it. The nature conduction of CNFs alone manages the conductivity of PCNFs when a high volume fraction of CNFs is used in polymer matrices, because the conductivity of polymer matrices is much lower than the conductivity of CNFs. Therefore, the direct relation between CNF conduction and the conductivity of PCNFs is logical. Additionally, it should be noted that the conductivity of CNFs is primarily based on waviness, defects, and size of nanofibers [77]. Conclusively, providing straight, long, and thin CNFs is important. However, very high levels of CNF conductivity remove its influence on the conductivity of PCNFs, confirming the advanced model.

**Figure 7:**
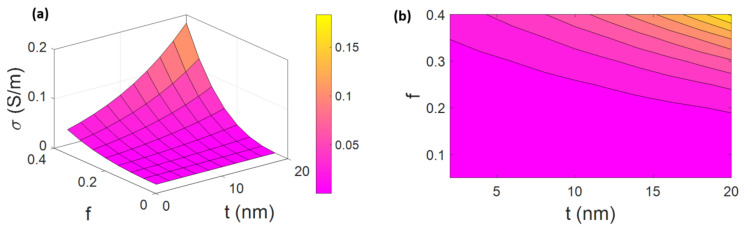
The relations of “t“ and “f “ with the conductivity by (**a**) 3D and (**b**) 2D illustrations.

Figure 7 illustrates the conductivity at different series of “t” and “f”. The best results of conductivity are achieved by the highest ranges of “t” and “f”, while the worst conduction is witnessed by the lowest levels of these terms. The highest and the lowest conductivities are estimated as 0.18 S/m (t = 20 nm and f = 0.4) and 0 (f < 0.19 at all ranges of t), respectively. Therefore, the effects of these factors on the conductivity are important, and the high values of interphase depth and network portion produce more conductivity.

As stated, an interphase layer around CNFs helps nanoparticles join to conductive nets before the actual physical links of CNFs in PCNFs. Clearly, percolated zones can be easily formed by a thick interphase. Based on Equation (2), a thicker interphase layer causes lower percolation onset, which can raise the conductivity in the composites. In other words, the interphase depth can positively influence the network by amplifying the effective CNFs [36,52,78]. In addition, perfect electron transfer needs a conductive CNF net in PCNFs. So, the density and dimensions of super-conductive nets influence the charge transfer and conduction of PCNFs. The size of contiguous conductive nets is directly shown by the fraction of percolated networks (f). An efficient electron transfer would not happen at the low levels of “f”, because nanofibers are not able to make a connection and nets. On the other hand, a higher “f” produces a denser network providing a higher conductivity. Accordingly, both interphase depth and network fraction reasonably handle the conductivity, validating the developed model.

**Figure 8:**
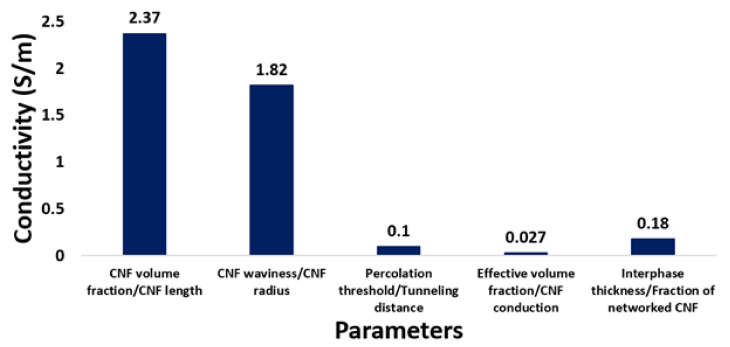
Variations of the calculated conductivity by different series of parameters.

Figure 8 shows the variations of conductivity by the mentioned factors based on the above discussion. The maximum variation of conductivity is obtained as 2.37 S/m by the volume fraction and length of CNF, while the minimum variation of conduction is achieved as 0.027 S/m by the effective volume fraction and conductivity of CNF. Additionally, optimal or desired electrical conductivity is calculated as 1.82 S/m at u = 1 and R = 10 nm, 0.1 S/m at $\varphi_{p}$ = 0.002 and d = 2 nm, and 0.18 S/m at t = 20 nm and f = 0.4. Based on these descriptions, CNF volume fraction and CNF length have the most significant influences on the conductivity.

### 3.2. Model Proofing by Experimented Data

**Figure 9:**
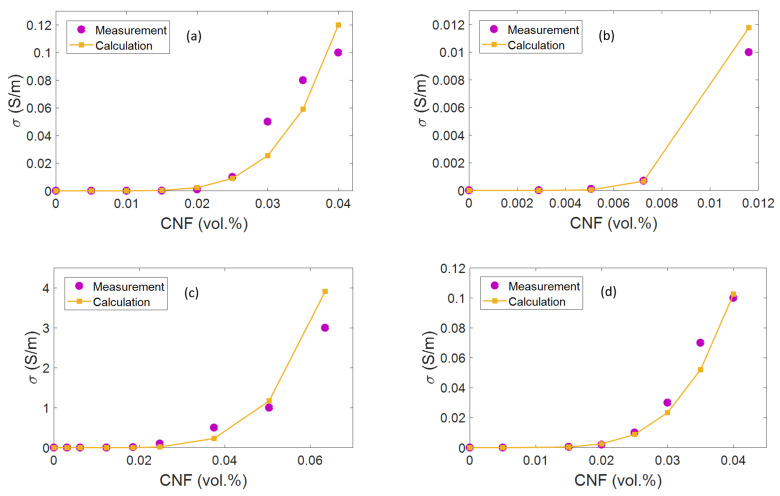
Agreement among theoretical and experimental data for (**a**) HDPE/CNF [79], (**b**) epoxy/randomly oriented CNF [80], (**c**) PMMA/CNF [81], and (**d**) HDPE/CNF [82] samples.

The validity of the advised model can be confirmed by the experimented results. The agreement of calculated and experimental results is demonstrated in Figure 9 for four samples. The experimental and theoretic calculations of “$\varphi_{p}$” are exhibited in Table 1. The theoretical estimations of percolation onset depict a good accordance with the experimental data, considering interphase depth in accordance with Equation (2). In addition, the thinnest interphase produces the highest level of percolation onset, whereas the smallest value of “$\varphi_{p}$” is produced by the thickest interphase around CNFs. In fact, the interphase region declines the “$\varphi_{p}$”and creates a contiguous network before the real joining of nanoparticles. Moreover, other terms such as CNF length, CNF radius, and CNF waviness can change the percolation level based on Equation (2). By comparing the suggested model to the experimented results, the tunneling distance is measured from 0.5 to 8 nm for the samples (Table 1). Conclusively, it is observed that the developed model can properly estimate the conductivity of PCNFs utilizing the experimental results and parametric analyses.

## 4. Conclusions

CNF waviness, filler dimensions, interphase region, effective volume fraction of filler, percolation onset, volume portion of networked CNFs, tunneling size, and CNF conduction were used to express a new methodology for the conductivity of PCNFs. The presented model showed the accurate and reasonable influences of all relevant factors on conductivity. Moreover, the experimental data of various samples agreed well with the calculations. These outcomes confirm the capability of the proposed model to forecast the conductivity of PCNFs. Conductivity is at a minimum with low levels of interphase thickness, CNF conduction, CNF radius, volume portion of percolated CNFs, effective volume fraction of CNFs, and CNF concentration, while maximum conductivity is achieved by the longest CNF lengths. Furthermore, lower percolation onset and shorter tunneling distance increase the conductivity. Additionally, the maximum conductivity is obtained as 2.37 S/m by a CNF volume fraction of 0.04 and a CNF length of 100 µm. However, effective volume fraction and conduction of CNFs have negligible impacts on the conductivity. It is stated that the high conductivity of CNFs cannot manipulate the conductivity of PCNFs.

## Figures and Tables

**Table 1:** Information of samples and theoretical and experimental values of percolation onset.

Samples	*R* (nm)	*u*	*l* (μm)	*t* (nm)	*d* (nm)	$\varphi_{p}\;\mathrm{Exp}.$	$\varphi_{p}\;\mathrm{Equation}\;(2)$
HDPE ^1^/CNF [79]	75	1.1	8.0	34	6.0	0.0100	0.0144
Epoxy/randomly oriented CNF [80]	67	1.1	20	30	0.5	0.0051	0.0058
PMMA ^2^/CNF [81]	65	1.3	100	5.0	1.0	0.0100	0.0107
HDPE/CNF [82]	75	1.2	8.0	35	8.0	0.0100	0.0113

^1^: high-density polyethylene; ^2^: poly (methyl methacrylate).

## Data Availability

The data that support the findings of this study are available on request.
